# Neutrophils and Activated Macrophages Control Mucosal Immunity by Proteolytic Cleavage of Antileukoproteinase

**DOI:** 10.3389/fimmu.2018.01154

**Published:** 2018-05-28

**Authors:** Jennifer Vandooren, Pieter Goeminne, Lise Boon, Estefania Ugarte-Berzal, Vasily Rybakin, Paul Proost, Ahmed M. Abu El-Asrar, Ghislain Opdenakker

**Affiliations:** ^1^Laboratory of Immunobiology, Department of Microbiology and Immunology, Rega Institute for Medical Research, University of Leuven, KU Leuven, Leuven, Belgium; ^2^Department of Respiratory Disease, University Hospital of Gasthuisberg, Leuven, Belgium; ^3^Department of Respiratory Disease, AZ Nikolaas, Sint-Niklaas, Belgium; ^4^Laboratory of Molecular Immunology, Department of Microbiology and Immunology, Rega Institute for Medical Research, University of Leuven, KU Leuven, Leuven, Belgium; ^5^Department of Ophthalmology and Dr. Nasser Al-Rashid Research Chair in Ophthalmology, College of Medicine, King Saud University, Riyadh, Saudi Arabia

**Keywords:** matrix metalloproteinases, proteases, antileukoproteinase, secretory leukocyte peptidase inhibitor, matrix metalloproteinase-9, neutrophils, macrophages, bronchiectasis

## Abstract

Antileukoproteinase or secretory leukocyte peptidase inhibitor is a small protein which protects the mucosal linings against excessive proteolysis, inflammation, and microbial infection. We discovered that gelatinase B or matrix metalloproteinase (MMP)-9, a secreted zinc-dependent endopeptidase typically found at sites of inflammation, destroys antileukoproteinase by cleavages within both of its two functional domains: the anti-microbial N-terminal and the anti-proteolytic C-terminal domains. Cleaved antileukoproteinase possessed a significantly lower ability to bind lipopolysaccharides (LPS) and a reduced capacity to inhibit neutrophil elastase (NE) activity. Whereas intact antileukoproteinase repressed proinflammatory transcript [prostaglandin-endoperoxide synthase 2 (*PTGS2*) and *IL6*] synthesis and protein secretion [e.g., of MMP-9] in human CD14^+^ blood monocytes stimulated with LPS, this effect was reduced or lost for cleaved antileukoproteinase. We demonstrated the *in vivo* presence of antileukoproteinase cleavage fragments in lower airway secretions of non-cystic fibrosis bronchiectasis patients with considerable levels of neutrophils and, hence, elastase and MMP-9 activity. As a comparison, other MMPs (MMP-2, MMP-7, and MMP-8) and serine proteases (NE, cathepsin G, and proteinase 3) were also able to cleave antileukoproteinase with similar or reduced efficiency. In conclusion, in specific mucosal pathologies, such as bronchiectasis, neutrophils, and macrophage subsets control local immune reactions by proteolytic regulation, here described as the balance between MMPs (in particular MMP-9), serine proteases and local tissue inhibitors.

## Introduction

Secretory leukocyte peptidase inhibitor (SLPI), also known as antileukoproteinase, is a small (107 AA) two-domain protein mainly produced by secretory cells lining the lungs, genitals, and digestive system (1, 2). At the mucosal lining, SLPI acts as a defense molecule against invading microorganisms by having antimicrobial, antiviral, and antifungal properties (3, 4). As its name suggests, SLPI also protects the mucosal epithelia against inflammatory damage by inhibiting proteolytic enzymes, in particular serine proteases, such as elastase, chymotrypsin, and cathepsin G (5). In contrast to circulating serine protease inhibitors such as α1-antitrypsin and the broad spectrum protease inhibitor α2-macroglobulin, SLPI can be considered as a local tissue protease inhibitor with limited systemic expression (6).

A second source of SLPI is inflammatory cells, including neutrophils, macrophages, and B-cells (7, 8). Indeed, besides the well-known anti-microbial and anti-proteolytic properties, SLPI has direct anti-inflammatory properties. At the molecular level, SLPI interferes with the cellular response of immune cells to lipopolysaccharides (LPS) (7). For example, SLPI is able to suppress (e.g., PTGS2) induction in human monocytes (9), to inhibit NF-κB by interference with the degradation of its cytosolic inhibitors IκBα and IκBβ (10) and to reduce the phagocytic capacity and respiratory burst of peripheral blood leukocytes upon stimulation (11). In addition, macrophages from SLPI KO mice have higher NF-κB activity, resulting in increased production of interleukin-6 and high-mobility group box-1 protein upon LPS stimulation. Together, these studies suggest an important role for SLPI in LPS-CD14-TLR4-mediated signaling pathways, prototypic for common infections with Gram-negative bacteria (8). These anti-inflammatory mechanisms likely form the basis of positive outcomes observed for treatment with SLPI in animal models for inflammatory diseases such as arthritis (11, 12) and healing of chronic wounds (6, 13). In addition, genetic deletion of SLPI in eosinophils and basophils results in increased allergic inflammation (14).

**Figure 1:**
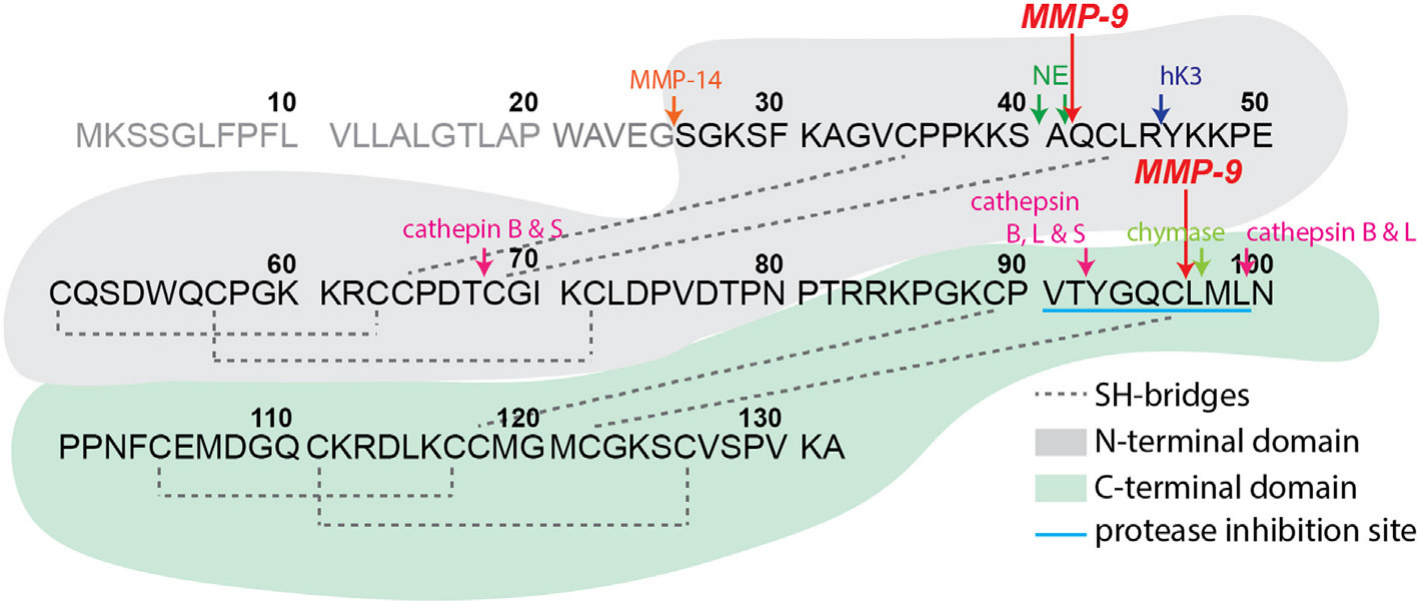
Secretory leukocyte peptidase inhibitor (SLPI) structure and proteolysis. Schematic representation of human SLPI (GenBank: CAA28187.1) structure, protein sequence, and experimentally confirmed protease digestion sites. SLPI contains two structurally similar domains, here colored in gray (N-terminal domain) and green (C-terminal domain) backgrounds. The C-terminal domain contains the main amino acid sequence for SLPI protease inhibition properties (sequence VTYGQCLML with blue underlining). Experimentally determined protease digestion sites are indicated by arrows and corresponding proteases marked above. The signal peptide sequence residues 1–25 are in gray. The experimental cleavage sites by matrix metalloproteinase-9 from the present study are indicated by large red arrows.

Structurally, SLPI has a boomerang-like shape with arms comprising two domains of similar architecture (5) (see Figure 1). While the N-terminal domain provides SLPI antimicrobial activity (4), the C-terminal domain is responsible for protease inhibition (15, 16). SLPI is rich in disulfide linkages, containing eight bridges in total (5), a feature which yields and fixes a specific folding pattern of both domains (see Figure 1) and is thought to protect the secreted SLPI from proteolysis. Nevertheless, several proteases have been reported to cleave SLPI, among which are serine proteases, including neutrophil elastase (NE) and proteinase 3 (17–19) and mast cell chymase (20) which are reciprocal targets for SLPI anti-proteolytic activity. In addition, cleavage products of SLPI have been found in inflammatory lung diseases and have been associated with SLPI-degrading proteases. For instance, cathepsin B, L, and S were found in emphysema (21), whereas NE was detected in *Pseudomonas*-infected cystic fibrosis lungs (17). Interestingly, most studies had a focus on a single protease or protease class, with broader interpretations of SLPI proteolysis missing.

In many ways protease research has become more complex than simple one-way protease–substrate–inhibitor interactions. This is especially clear within the field of MMPs. For example, MMPs cleave cytokines, thereby activating or inactivating cytokine activities and, respectively, increasing or decreasing the expression of other proteases (22, 23). In addition, proteases are able to cleave and inactivate inhibitors of MMP activity such as in processing of tissue inhibitors of metalloproteinases (TIMPs) by NE (24). Proteases even activate MMPs by removal of proMMP inhibitory prodomains, as observed during activation of proMMP-9 by NE (25). These interactions reveal the existence of a complex and highly dynamic interconnected protease network, also referred to as the “protease web” (26, 27).

Comparable to SLPI, MMP-9, also called gelatinase B, is produced and secreted by immune cells, in particular by neutrophils, and attributed antimicrobial and immune-modulatory functions (28). In addition, MMP-9 is implicated in many chronic lung pathologies, including bronchiectasis (29) and cystic fibrosis (30, 31). We recently discovered the *slpi* gene to be one of the only three genes significantly altered in MMP-9 KO mice compared to WT mice (32). In addition, others found that SLPI-deficient eosinophils have increased MMP-9 gene transcription (14). These and other studies point toward reciprocal regulation of SLPI and MMP-9 and impose to further investigate a possible link between MMP-9 and SLPI. We showed that MMP-9 was able to cleave SLPI both in its N-terminal (anti-microbial) and C-terminal (anti-proteolytic) domains. These cleavages resulted in the loss of both SLPI anti-proteolytic and anti-inflammatory activities. In sputum samples from bronchiectasis patients with high levels of active MMP-9 and NE activity, we detected SLPI cleavage fragments. Finally, we extended SLPI cleavage to other neutrophil-derived MMPs and serine proteases, indicating a specific relevance for the location of proteases and their inhibitors in disease states. Our data are significant for neutrophil-mediated diseases, in particular, at mucosal linings.

## Materials and Methods

### Proteins, Reagents, and Buffers

Lipopolysaccharides from *Escherichia coli* 0111: B4 (L4391) was purchased from Sigma-Aldrich (St. Louis, MO, USA). Recombinant human proMMP-2 (CHO cell-derived), proMMP-3 (NS0 cell-derived), proMMP-7 (NS0 cell-derived), and proMMP-8 (NS0 cell-derived) were purchased from R&D systems (Minneapolis, MN, USA), dissolved in assay buffer (150 mM NaCl, 5 mM CaCl_2_, 0.01% Tween-20, 50 mM Tris, pH 7.4) to a concentration of 100 µg/ml and activated by incubation with 1 mM p-aminophenylmercuric acetate for, respectively, 1h, 6h, 2h and 1h. Recombinant human full-length proMMP-9 (92 kDa) was expressed in Sf9 insect cells, purified by gelatin-Sepharose chromatography and activated by incubation with the catalytic domain of stromelysin-1/MMP-3 (cat. No. 444217, Merck Millipore, Darmstadt, Germany) as previously described (33). Activation of all MMPs was confirmed by a band shift of approximately 10 kDa, corresponding to the removal of the propeptide domain. Active human NE was purchased from Abcam (Cambridge, UK), human neutrophil proteinase-3 isolated from human sputum neutrophils, and human neutrophil cathepsin G was purchased from Enzo Lifesciences (Brussels, Belgium). Human SLPI was purchased from R&D systems (Minneapolis, MN, USA). SLPI digestions by MMPs were performed in assay buffer (150 mM NaCl, 5 mM CaCl_2_, 0.01% Tween-20, 50 mM Tris, pH 7.4). SLPI digestion experiments with NE, cathepsin G, and proteinase-3 were performed in 200 mM Tris, pH 8.8.

### NE Inhibition Experiments

Neutrophil elastase activity was measured in the presence or absence of intact SLPI or SLPI treated with active MMP-9. For the gelatin degradation assay, NE was used at a final concentration of 5 nM and activity was monitored immediately after addition of 2.5 µg/ml DQ™-gelatin (Invitrogen, Carlsbad, CA, USA). To avoid background gelatinolysis by MMP-9, derived from the digestion of SLPI, all reactions were performed in the presence of 50 µM SB-3CT (Santa Cruz Biotechnology, Dallas, TX, USA). For a second activity test, we used the fluorogenic elastase substrate V (MeOSuc-Ala-Ala-Pro-Val-AMC) (Millipore, Burlington, MA, USA). 5 nM NE was combined with different concentrations and digestions of SLPI and incubated for 30 min at 37°C. Next, 20 µM elastase V substrate was added and fluorescence was measured every minute. Data derived from both experiments were fitted by linear regression and the velocity of the reaction was used as a measure for enzyme activity.

### SDS-PAGE, Western-Blot Analysis, Edman Degradation, and Gelatin Zymography Analyses

Samples were chemically reduced and buffered and proteins were separated on 16% Novex Tris-glycine gels in a mini gel tank as instructed by the supplier (Invitrogen, Carlsbad, CA, USA). Next, proteins were either stained directly or transferred for further processing. Direct staining of the proteins was achieved by Coomassie Brilliant Blue staining or with the SilverQuest™ Silver Staining Kit (Invitrogen, Carlsbad, CA, USA). Transfer to PVDF membranes was done using the Trans-Blot Turbo Transfer System with associated materials and protocols (Biorad, Hercules, CA, USA). For Edman degradation analysis, PVDF membranes were briefly washed with water, stained for 1 min (0.1% Coomassie Brilliant Blue, 1% acetic acid, 40% methanol), and destained in 40% methanol. After drying the membrane, protein bands were excised and N-terminal Edman sequencing was performed by Alphalyse (Denmark). For Western blot analysis, PVDF membranes were first blocked for 1 h in 5% BSA with TBST buffer (150 mM NaCl, 0.1% Tween 20, 50 mM Tris, pH 7.5). Next, the membranes were incubated overnight with goat anti-hSLPI (AF1274, R&D Systems, Minneapolis, MN, USA), mouse anti-hNE (MAB91671, R&D Systems, Minneapolis, MN, USA), or mouse anti-hMMP-9 [REGA-3G12, see Ref. (34)]. After washing, the blot was incubated with peroxidase-conjugated anti-goat IgG (PI-9500, Vector Labs, Burlingame, CA, USA) or anti-mouse IgG (115-035-071, Jackson ImmunoResearch, PA, USA) for 1 h at room temperature. Finally, Western blots were imaged using the Vilber Lourmat Fusion system (Labtech International, Heathfield, TN, USA) and Pierce ECL Western Blotting Substrate (Thermo Fisher Scientific, Waltham, MA, USA). Gelatin zymography gels were prepared consisting of a 7.5% acrylamide separating gel with 1 mg/ml gelatin (Sigma Aldrich G1890), topped with a 5% stacking gel. Gels were placed in an electrophoresis system with running buffer (25 mM Tris, 192 mM glycine, 0.1% SDS) and samples, prepared in non-reducing loading dye, were added. After electrophoretic protein separation, the gels were washed twice for 20 min in re-activation solution (2.5% Triton-X-100). Next, the gel was incubated overnight in 10 mM CaCl_2_ and 50 mM Tris–HCl, pH 7.5 at 37°C. Staining was performed with 0.1% Coomassie Brilliant Blue R-350 (GE Healthcare) and zymograms were analyzed densitometrically using the ImageQuant TL software (GE Healthcare).

### LPS-Binding ELISA

Nunc Maxisorp plates were coated with 100 ng LPS per well (in RPMI 1640) and incubated at 37°C for 3 h. After a brief wash with Milli-Q water, the plate was allowed to air-dry overnight. Next, the plate was blocked with 1% BSA in PBS for 2 h. After three cycles with wash buffer (0.05% Tween 20 in PBS, pH 7.4) different dilutions of SLPI in RPMI 1640 were added to the wells and allowed to react for 2 h. Three more washes were performed and anti-SLPI-biotin (1/1,000 in 1% BSA in PBS) (cat. no. BAF1274, R&D Systems, Minneapolis, MN, USA) was added to the wells. The plates were incubated for 2 h at room temperature, washed three times, and streptavidin peroxidase (1/2,500 in 1% BSA in PBS) was added. After 20 min and a final wash step, the color reagents were added (1/1 hydrogen peroxide/tetramethylbenzidine). The color reaction was stopped with 50 µl of 2N sulfuric acid and absorbance was measured at 450 nm.

### Human Monocyte Purification and Stimulation

Peripheral blood mononuclear cells were purified from the blood of healthy donors by density gradient centrifugation on Ficoll-sodium diatrizoate (Lymphoprep, Axis-Shield PoC AS, Oslo, Norway). Next, CD14^+^ monocytes were isolated using the EasySep™ human CD14 positive selection kit (StemCell, Grenoble, France). Cells were seeded in 24-well plates at a density of 2 × 10^6^ cells/ml and SLPI or SLPI digests were added at a concentration of 1 µg/ml. After 30 min incubation at 37°C, cells were stimulated with 1 µg/ml LPS from *Escherichia coli* 0111: B4 (L4391, Sigma-Aldrich, St. Louis, MO, USA). After 24 h, cells and supernatants were collected.

### RNA Extraction and RT-qPCR

RNA was extracted from human monocytes using the Qiagen RNeasy mini kit (Qiagen). The quantity and quality of the extracted RNA was examined using the CLARIOstar with LVis Plate (BMG Labtech, Ortenberg, Germany). RNA was converted to cDNA with a high-capacity cDNA reverse transcription kit (Applied Biosystems) and a GeneAmp PCR system 9700 (Applied Biosystems). Finally, qPCR was performed using TaqMan^®^ fast universal PCR master mix (Applied Biosystems), PrimeTime^®^ predesigned qPCR assays (IDT), and a 7500 Fast Real-Time PCR System (Applied Biosystems). The following genes were analyzed: *IL6* (Hs.PT.58.40226675), *IL8* (Hs.PT.58.39926886.g), *PTGS2* (Hs.PT.58.77266), and *GAPDH* (Hs.PT.39a.22214836). *GADPH* was used as a housekeeping gene.

### Patient Samples and Sputum Gelatinolytic Activity Test

Samples from non-cystic fibrosis bronchiectasis (NCFB) patients were collected at the University Hospital Leuven, Belgium. This study was carried out in accordance with the recommendations of the ethical committee for research at UZ/KU Leuven, CME. The protocol was approved by the CME under license (B51060-B32220084152). All subjects gave written informed consent in accordance with the Declaration of Helsinki. Sputum production was induced by inhalation of hypertonic saline 5% and sputum was collected and processed as described previously (29). A detailed report of patient characteristics can be found in Ref. (29). The total protein content of the sputum samples was determined with the Pierce BCA Protein Assay Kit (cat. no. 23225, Thermo Fisher Scientific, Waltham, MA, USA). To measure gelatinolytic activity, sputum samples at the equivalent of 300 µg total protein were added to a 96-well plate. To inhibit NE, an inhibitor (Elastase Inhibitor IV, Calbiochem) was added to a final concentration of 10 µM and the plate was incubated for 30 min at 37°C. Finally, 2.5 µg/ml DQ™-gelatin (Invitrogen, Carlsbad, CA, USA) was added to a final volume of 100 µl and the increase in fluorescence was measured every 2 min.

### Statistics

All data were analyzed with the GraphPad Prism 7 software. Dose-response curves were constructed by using four-parameter dose response fits and in parallel analyzed by extra sum-of-squares F tests to compare whether the datasets differed significantly. Data obtained for human monocyte stimulations were analyzed pairwise, since each experiment was performed with monocytes from a different donor and thus matched for each experiment (Friedman test). Data obtained from patient samples are represented as median with 25–75% interquartile range and differences were statistically compared using a Kruskal–Wallis test.

## Results

### MMP-9 Cleaves Human SLPI

**Figure 2:**
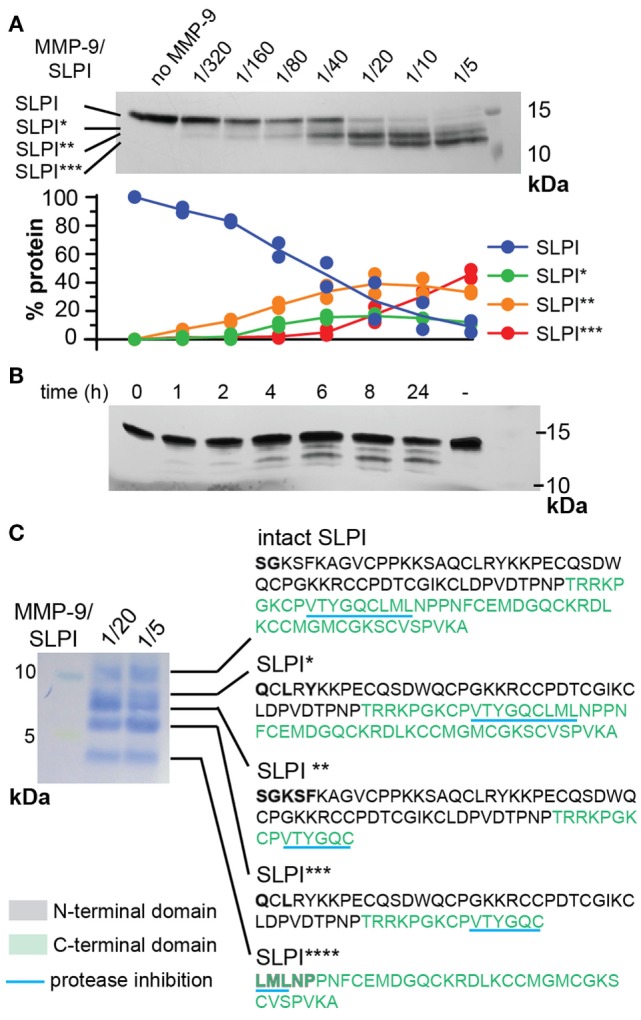
Cleavage of human secretory leukocyte peptidase inhibitor (SLPI) by matrix metalloproteases (MMP-9). **(A)**; Incubation of SLPI with increasing quantities of active MMP-9 for 24 h at 37°C. Aside intact SLPI, three SLPI fragments were visualized; SLPI*, SLPI**, and SLPI*** (top). Densitometry analysis (bottom) of two independent experiments illustrates the appearance of fragments in the following order: SLPI**, SLPI*, and SLPI***. Data expressed as percentage of total SLPI protein. **(B)** Time-dependent incubation of SLPI with active MMP-9 (molar ratio 1/20). **(C)** Protein staining after blot transfer to a solid support and N-terminal sequencing of SLPI fragments. After blotting of larger quantities of SLPI, four cleavage fragments (SLPI*–SLPI****) were visible. N-terminal sequencing analysis revealed the presence of two MMP-9 digestion sites, also indicated in Figure 1. Amino acids retrieved by N-terminal sequencing are marked in bold. Amino acid color codes correspond with the N-terminal and C-terminal domains, as shown in Figure 1.

Based on our previous data showing that *slpi* is one of only three gene transcripts significantly increased in mucosal tissues from MMP-9 KO mice compared to WT mice (32), we questioned whether SLPI might be a substrate for MMP-9. Therefore, we first performed *in silico* analysis of potential digestion sites by using the protease specificity prediction server (35). Eight potential SLPI proteases were retrieved (see Figure S1 in Supplementary Material). Two proteases (HIV-1 protease and glutamyl endopeptidase I) were from microbial origin and one (thylakoidal processing peptidase) from plant origin. The remaining proteases were either matrix metalloproteases (MMP-2, -3, and -9) or serine proteases (NE and cathepsin G) with MMP-9 having the highest score. In addition, the tool predicted a cleavage site for multiple protease families around Glu^106^. Next, we investigated whether human SLPI was cleaved by activated recombinant human MMP-9 *in vitro*. SLPI was incubated with different amounts of active MMP-9 for 24 h. Depending on the MMP-9 concentration, SLPI was converted into three bands which we labeled SLPI*, SLPI**, and SLPI*** in order of decreasing fragment size (Figure 2A). By quantification of the protein bands we found that the most efficiently produced fragment is SLPI** (starting at the lowest MMP-9/SLPI molar ratio of 1/320), followed by SLPI* (1/80), and finally SLPI*** (1/40). In addition, during time-course experiments, SLPI** was generated as early as 1 h after incubation (Figure 2B).

### Both N-Terminal and C-Terminal Domains of SLPI Are Cleaved by MMP-9

To elaborate on potential functional implications we determined the exact cleavage sites. To cover all SLPI fragments, two preparations of cleaved SLPI were prepared; MMP-9/SLPI ratio 1/20 (efficient cleavage sites) and 1/5 (less efficient cleavage sites). Blotting and subsequent staining of larger amounts of SLPI preparations revealed an additional SLPI cleavage band smaller than 5 kDa (Figure 2C), which we designated SLPI****. Subsequently, N-terminal sequencing analysis revealed two cleavage sites. The first digestion site presented in the N-terminal domain between Ala^41^ and Gln^42^, and overlapped with a previously predicted cleavage site for NE (17) (Figure 1). The second site was within the C-terminal domain between Cys^96^ and Leu^97^, located exactly within the amino acid sequence responsible for protease inhibition (Figure 1). Finally, by combining sequencing data and predicted molecular weights of the fragments, the nature of SLPI*–SLPI**** could be determined (Figure 2C). Interestingly, since the generation of fragment SLPI** appears most efficiently, this indicates that the C-terminal cleavage within the protease inhibition site is executed first, followed by N-terminal cleavage. In addition, although SLPI cleavage by MMP-9 was predicted *in silico*, the exact location of these cleavage sites did not correspond with the experimentally determined ones.

### MMP-9 Reduces the Capacity of SLPI to Inhibit NE Proteolytic Activity

**Figure 3:**
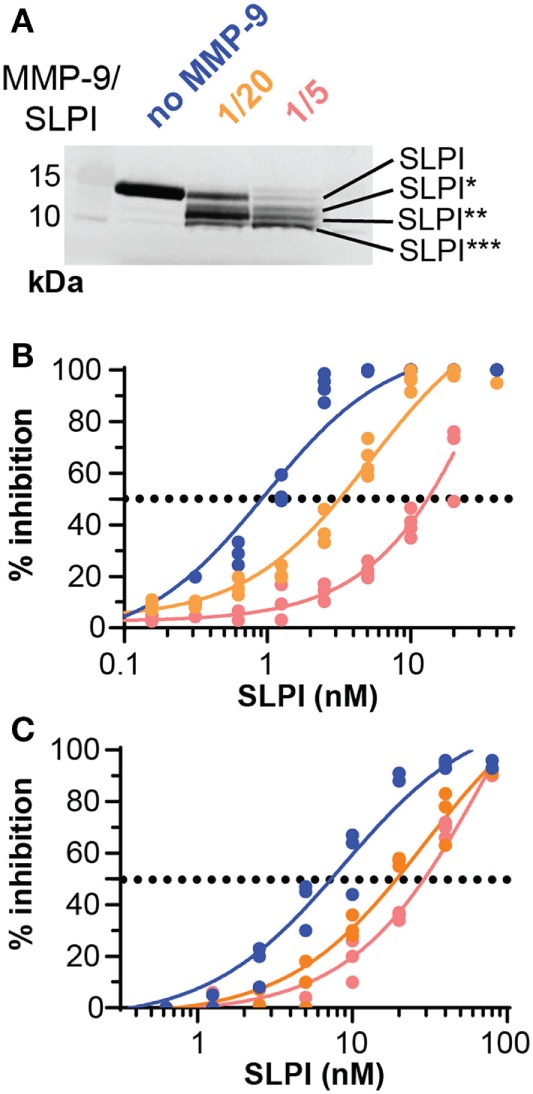
Proteolysis of secretory leukocyte peptidase inhibitor (SLPI) by matrix metalloprotease-9 (MMP-9) reduces antiproteolytic activity against neutrophil elastase (NE). **(A)** Digestion of SLPI at 1/20 and 1/5 molar ratio of MMP-9/SLPI. Representative image of several batches (Figure S2 in Supplementary Material). **(B)** Percentage inhibition of NE gelatinolytic activity by SLPI (blue), MMP-9/SLPI 1/20 (orange), and MMP-9/SLPI 1/5 (red). Data fitted with a four-parameter dose-response fit, *n* = 3–4. **(C)** Percentage inhibition of NE activity to degrade a small fluorogenic NE substrate by SLPI (blue line), MMP-9/SLPI 1/20 (orange line), and MMP-9/SLPI 1/5 (red line). Data fitted with a four-parameter dose-response fit, *n* = 3.

We evaluated the functional effect of cleavage by MMP-9 at two enzyme substrate ratios: MMP-9/SLPI 1/20 (efficient cleavage sites) and 1/5 (including also processing at less efficient cleavage sites). Although some batch-to-batch variability existed (see Figure S2 in Supplementary Material), the 1/20 reaction products mainly contained fragments with C-terminally cleaved SLPI (SLPI**), whereas the 1/5 reactions resulted in more SLPI with both N-terminal and C-terminal cleavages (SLPI***) (Figure 3A). Since MMP-9 efficiently cleaves the C-terminal anti-proteolytic site which accounts for most of the SLPI anti-proteolytic activity against serine proteases, in particular NE (36, 37), we hypothesized that MMP-9 cleaved SLPI might lose its anti-proteolytic activity. SLPI anti-proteolytic activity on NE was measured using two activity tests: the degradation of fluorogenic gelatin (Figure 3B) and the degradation of a NE-specific small fluorogenic substrate (Figure 3C). Dose response curves for NE inhibition by SLPI and cleaved-SLPI were fitted and IC_50_ values were calculated (Table 1). Statistical comparisons of the best-fit IC_50_ values (extra sum-of-squares F test) confirmed that the residual inhibitory activities of SLPI were significantly different (*p* < 0.0001) for each dataset and this for both substrates. Indeed, the ability of cleaved SLPI to inhibit NE activity was considerably less than that of intact SLPI. In addition, the effect increased depending on the extent of SLPI processing.

**Table 1:** Inhibitory capacity of secretory leukocyte peptidase inhibitor (SLPI) and matrix metalloproteinase-9 (MMP-9)-treated SLPI on neutrophil elastase.

	SLPI	Matrix metalloproteases (MMP)-9/SLPI (1/20)	MMP-9/SLPI (1/5)
	**Gelatin degradation**		

IC_50_ (CI)	0.97 (0.74–1.27) nM	5.24 (3.94–6.99) nM	51.18 (22.92–587.1) nM
*R*^2^	0.959	0.962	0.954
*n*	4	4	4

	**Peptide degradation**		

IC_50_ (CI)	8.71 (6.53–11.67) nM	28.92 (21.48–39.76) nM	76.32 (52.81–118.5) nM
*R*^2^	0.967	0.973	0.974
*n*	3	3	3

*Half-maximal inhibitory concentration (IC_50_), reported as best-fit value and 95% confidence interval (CI). *R*^2^, goodness of dose-response fit (three parameters); n, number of experiments*.

### MMP-9 Diminishes the Capacity of SLPI to Bind LPS

**Figure 4:**
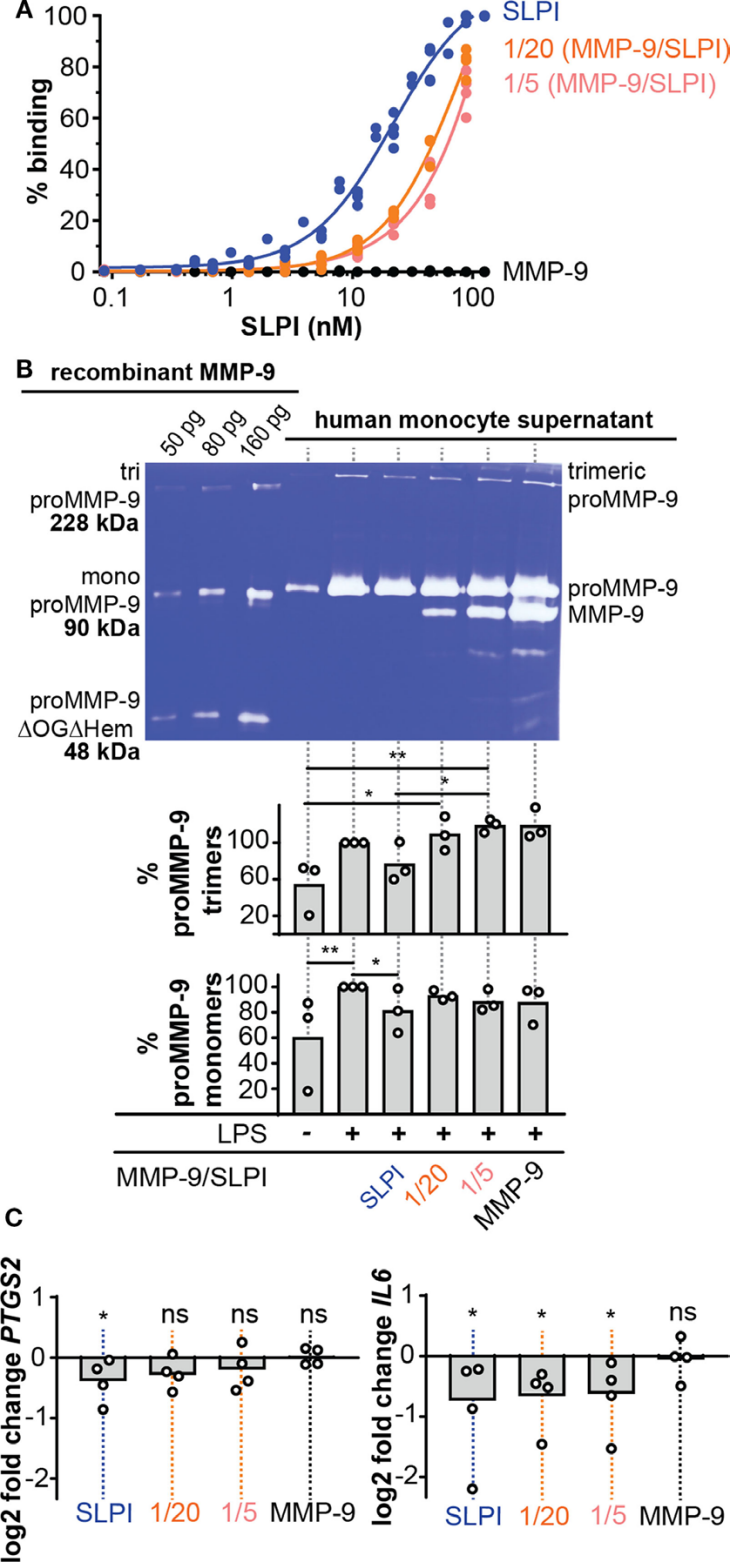
Lipopolysaccharide (LPS)-binding and anti-inflammatory properties of cleaved-SLPI. **(A)**, Analysis of LPS-binding capacity of secretory leukocyte peptidase inhibitor (SLPI) (blue), 1/20 cleaved-SLPI [matrix metalloproteinase-9 (MMP-9/SLPI)] (orange), and 1/5 cleaved-SLPI (MMP-9/SLPI) (red) by LPS-binding ELISA. *n* = 3, statistical comparison of best fits by extra sum-of-square F test indicated that the SLPI curve is significantly different from the 1/20 and 1/5 condition. **(B)** Zymography analysis of culture supernatant from human monocytes treated with SLPI or cleaved SLPI prior to stimulation with LPS (1 µg/ml). Control conditions included monocytes with/without LPS and treated with matrix metalloproteases (MMP)-9 at the same concentration as the 1/5 MMP-9/SLPI condition. No variation in cell numbers was observed due to the stimuli (see Figure S3 in Supplementary Material). Top, representative zymography image. Bottom, densitometry analysis of three independent experiments. Data shown as the percentage of proMMP-9 monomer and trimer levels in supernatants from cultured cells compared to cell only stimulated with LPS. *P < 0.05, **P < 0.01, as determined by Friedman test with uncorrected Dunn’s test (*n* = 3). **(C)** Analysis of *PTGS2* and *IL6* RNA expression in human monocytes treated with SLPI or cleaved SLPI (1/5 and 1/20) prior to stimulation with LPS (1 µg/ml). Each data point represents an independent experiment with a fresh and different pool of human monocytes. Data represented as log2 fold change compared to cells stimulated only with LPS. *P < 0.05, as determined by Friedman test with uncorrected Dunn’s test for the indicated condition compared to cell only stimulated with LPS.

Besides having a protease inhibition site, SLPI is also a small cationic protein with antimicrobial “defensin-like” and anti-inflammatory features (3). One mechanism through which this is executed is by direct binding to LPS, thereby interfering with CD14-LPS binding and subsequent uptake by macrophages (16, 38, 39). We investigated whether SLPI digestion by MMP-9 affected LPS-binding properties, as previously shown for NE-dependent SLPI cleavage (17). While intact SLPI bound LPS in a dose-dependent manner, MMP-9-treated SLPI had reduced LPS-binding capacity (Figure 4A). This effect was most pronounced for MMP-9/SLPI 1/20, with little change upon further SLPI processing (MMP-9/SLPI 1/5). This was also confirmed by statistical comparison of the best fit parameters (extra sum-of-square F test), where SLPI and MMP-9-treated SLPI preferentially fitted different curves, while 1/20 and 1/5 (MMP-9/SLPI) could also be fitted by one single curve.

### MMP-9 Secretion by LPS-Challenged Human Monocytes Is Reduced Only by Intact SLPI

To analyze the detrimental effect of MMP-9 cleavage on SLPI anti-inflammatory properties, we performed experiments with human monocytes, purified from fresh human blood by positive selection for CD14. Next, cells were pre-treated with SLPI or cleaved-SLPI and stimulated with LPS. Since MMP-9 production by monocytes is directly regulated by NF-κB, a downstream transcription factor of pro-inflammatory stimuli (28) and to further elaborate on potential feedback mechanisms, we first evaluated changes in MMP-9 production. Cell culture supernatants were analyzed by gelatin gel zymography (40) with the use of a standard mixture of three human recombinant MMP-9 forms [including MMP-9 trimers, monomers, and a deletion mutant lacking the O-glycosylated and hemopexin domain (proMMP-9ΔOGΔHem)] as molecular weight markers (Figure 4B). While LPS clearly stimulated MMP-9 production, human monocytes pretreated with intact SLPI secreted lower amounts of proMMP-9 upon LPS stimulation (Figure 4B). This effect was visible for both monomeric proMMP-9 and trimeric MMP-9 (41). Densitometric analysis of three experiments with three batches of independently purified monocytes from different donors showed significant differences in the presence or absence of intact SLPI. However, this effect was lost when SLPI was cleaved with MMP-9 prior to stimulation. Interestingly, these data indicate a proteolytic feedback loop in the presence of LPS, because SLPI cleavage by MMP-9 might favor production of MMP-9.

### MMP-9 Abolishes SLPI Anti-Inflammatory Activity

While the effect on MMP-9 secretion (as mentioned above) already proved the occurrence of changes in the anti-inflammatory action of SLPI, we reinforced these findings by analyzing the transcription of other markers of inflammation (Figure 4C). Therefore, we pre-exposed LPS-stimulated human monocytes to intact SLPI or cleaved-SLPI and performed RT-qPCR analysis. In line with previous publications (8, 9), transcription of both the mouse *PTGS2* and *IL6* genes was reduced with intact SLPI, as here shown for human monocytes. Interestingly, this effect was reduced for MMP-9-treated SLPI and this was most pronounced for *PTGS2*. Although RT-qPCR analysis of *IL8* presented with a similar trend, no significant differences were found, likely due to the fact that MMP-9 is also able to modify IL-8 (22) (Figure S3C in Supplementary Material).

### SLPI Fragments Are Found in Airways of Bronchiectasis Patients With Increased MMP-9 and NE Activity

**Figure 5:**
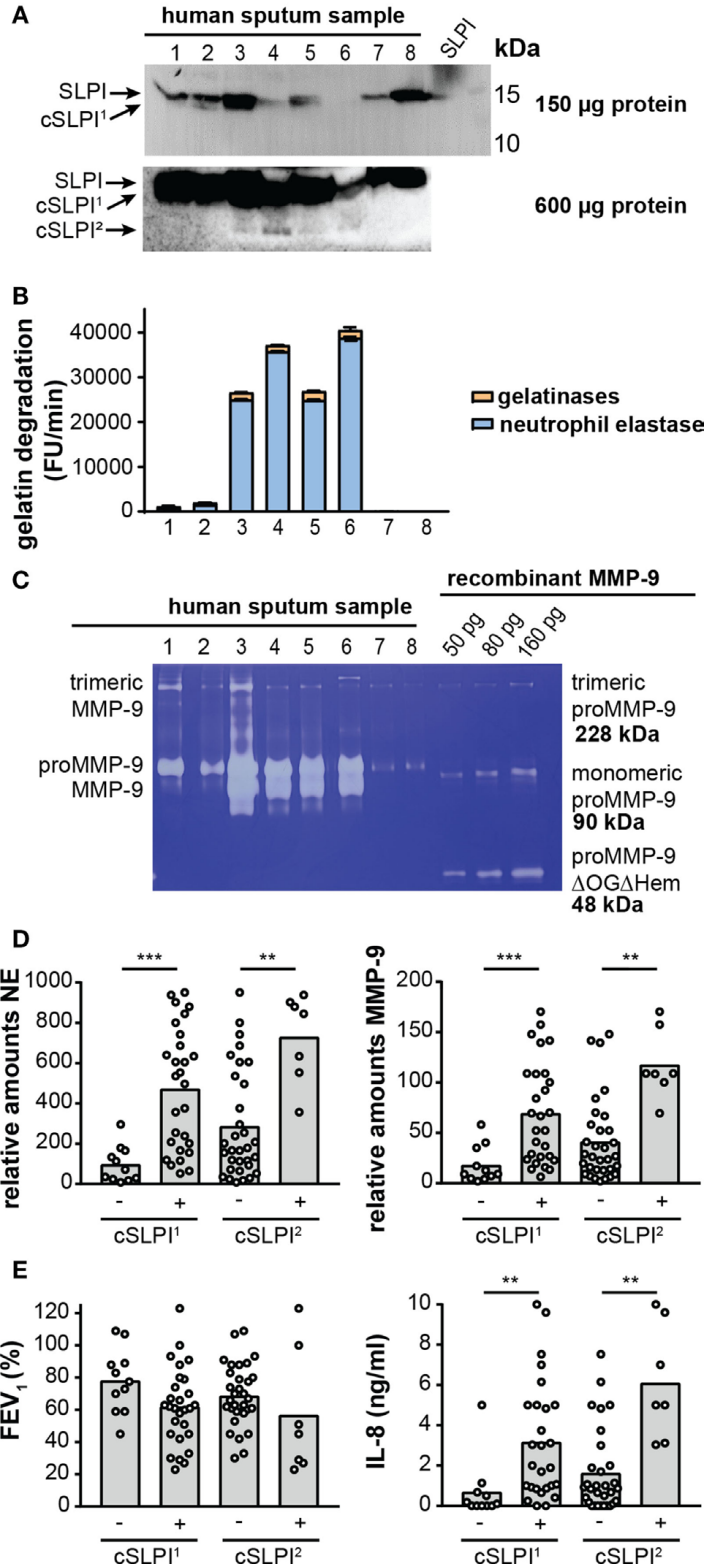
Secretory leukocyte peptidase inhibitor (SLPI) fragments and protease activity in lower airway secretion from patients with non-cystic fibrosis bronchiectasis. **(A)** Western blot analysis of sputum samples from eight representative bronchiectasis patients with anti-SLPI. Top image, total loading volume of each sample corresponds to 150 µg total protein. Bottom image, total loading volume corresponds to 600 µg total protein. Depending on the loading quantity, different SLPI cleavage fragments, here designated cSLPI^1^ and cSLPI^2^, were retrieved. Representative images of two experiments per condition. A full scan of the entire original Western blots can be found in Figure S5 in Supplementary Material and a full analysis of samples from 40 patients can be found in Figure S4 in Supplementary Material. **(B)** Total gelatinolytic activity in lower airway secretions of eight representative samples. Loading equivalent to 300 µg total protein. Efficiency of the gelatin degradation reaction expressed as the increase in fluorescence units (FU) per minute. Total bar height indicates the total gelatinolytic activity. The blue portion indicates the proportion of gelatinolytic activity derived from neutrophil elastase (NE), as determined by addition of an elastase inhibitor. The experiment was performed three times. **(C)** Gelatin zymography analysis of eight representative sputum samples equivalent to 6.5 µg total protein. Left, indication of trimeric MMP-9, proMMP-9, and activated MMP-9. Right, different concentrations of recombinant proMMP-9 as a measure for protein quantity and molecular weights. Indication of trimeric proMMP-9 (41), monomeric proMMP-9, and a low-molecular weight proMMP-9 domain deletion mutant lacking the O-glycosylated and hemopexin domains (proMMP-9 ΔOGΔHem). Due to less glycosylation, recombinant proMMP-9 typically appears at a slightly lower molecular weight than human proMMP-9. Representative image of four zymography experiments. **(D)** Relative amounts of NE and MMP-9 in samples with or without cSLPI^1^ and cSLPI^2^ as determined by Western blot analysis (see Figure S5 in Supplementary Material for Western blot images). **P < 0.01, ***P < 0.001, as determined by Kruskal–Wallis test. **(E)** Forced expiratory volume in 1 s (FEV_1_) and IL-8 protein levels in samples with or without cSLPI^1^ and cSLPI^2^. The patient cohort was described in Ref. (29). **P < 0.01, as determined by Kruskal–Wallis test.

Recently, we reported high proteolysis levels in sputum samples from NCFB patients. In particular, we showed that samples from the lower airways were rich in neutrophils and presented high proteolytic activity resulting from a combination of NE activity (±80%) and gelatinase (MMP-2 and MMP-9) activity (±20%) (29). Since others have reported lower SLPI concentrations and SLPI fragments in chronic obstructive pulmonary disease (42), emphysema (21), and cystic fibrosis (17, 18), we questioned whether such fragments are present in the airways of bronchiectasis patients, and, how these fragments relate to protease levels and activity. In a first exploration, 40 sputum samples from non-cystic fibrosis bronchiectasis patients were analyzed by Western blot with anti-SLPI antibodies (see Figure S4A in Supplementary Material). Three SLPI patterns were observed: a strong intact SLPI signal without cleaved SLPI (cSLPI), a weak SLPI signal but no cSLPI and a weak SLPI signal with visible cSLPI. To allow more accurate analysis, the total protein content of each sample was determined and sample loading volumes were adjusted accordingly. Western blot analysis of the equivalent of 150 µg protein (Figure 5A; Figure S4B in Supplementary Material) revealed a first cleaved SLPI form (SLPI^1^). To search for additional cleavage fragments, we next loaded a high amount of protein (600 µg, Figure S4C in Supplementary Material) and were able to retrieve an additional cleavage band (cSLPI^2^), showing that SLPI is indeed cleaved in the airways of bronchiectasis patients and that cleavage fragments can be found.

Next, we examined whether these fragments correlated with the proteolytic environment. Interestingly, with the use of the gelatin degradation assay we revealed higher gelatinolytic activity in samples with cSLPI (Table 2). Since net gelatinolytic activity in neutrophil-rich samples is a combination of NE and gelatinase (MMP-2 and MMP-9) activities (43), we repeated the gelatin degradation assay in the presence of an elastase inhibitor. As expected, a combination of gelatinase and NE activity was found and higher percentages of NE corresponding to samples containing the cSLPI^1^ fragment (Table 2; Figure 5B). Typically, MMP-9 is secreted by cells as an inactive zymogen with a propeptide domain which blocks the active site (designated as proMMP-9). Activation of proMMP-9 requires the proteolytic removal of the propeptide (by other proteases including MMP-3 and NE) or modification of the inhibitory cysteine in the propeptide domain (28). Since proteolytic activation of proMMP-9 is visible as a molecular weight shift of approximately 10 kDa, we performed gelatin zymography analysis to establish the presence of activated MMP-9 (40). Increased amounts of activated MMP-9 were found in samples with the SLPI^1^ cleavage fragments (Table 2; Figure 5C). Finally, we performed Western blot analysis of all samples (Figure S4B in Supplementary Material) and confirmed the presence of NE and MMP-9. Relative amounts of both NE and MMP-9 increased with sputum purulence (Figure S6 in Supplementary Material) as previously reported (29) and were significantly increased in samples containing cSLPI^1^ and cSLPI^2^ (Figure 5D). Although we were technically unable to directly match SLPI cleavage fragments to NE or MMP-9, based on our correlations we suggest a combined effect of NE and MMP-9 in the formation of cleaved SLPI. SLPI cleavage fragments were further analyzed in function of sputum microbiology, disease severity, or inflammatory markers. While no significant differences in bacterial colonization were found between patients with or without SLPI fragments, a trend was seen for lower FEV_1_ levels in patients with SLPI fragments. In addition, samples with SLPI fragments contained significantly higher levels of IL-8 (Figure 5E; Table 2), a potent neutrophil chemoattractant, and a granulocytosis-promoting protein which triggers neutrophils to release the content of their granules including MMP-9 (44).

**Table 2:** Detailed analysis of lower airway secretion from patients with non-cystic fibrosis bronchiectasis.

	No cSLPI^1^ (*n* = 11)	cSLPI^1^ (*n* = 28)	*P*-value	No cSLPI^2^ (*n* = 32)	cSLPI^2^ (*n* = 7)	*P*-value
Total gelatinolytic activity (pM)^a^	50 (0–50)	200 (10–500)	0.0399*	50 (10–500)	2,000 (100–5,000)	0.0192*
% NE activity^a^	0 (0–0)	35 (0–83)	0.0574^ns^	0 (0–77)	77 (69–90)	0.0103*
% Activated MMP-9^a^	7 (0–20)	23 (9–32)	0.0265*	11 (0–29)	28 (11–32)	0.2405^ns^
Relative NE content^b^	71 (24–165)	516 (175–728)	0.0003***	172 (77–526)	844 (553–902)	0.0031**
Relative MMP-9 content^b^	9 (5–35)	59 (24–109)	0.0010***	26 (13–57)	109 (100–157)	0.0011**
FEV_1_ (%)	78 (89–59)	62 (45–78)	0.1187^ns^	67 (59–82)	45 (27–100)	0.2616^ns^
IL-8 (ng/ml)^a^	0.1 (0–0.7)	2 (0.9–5)	0.0026**	0.9 (0.1–2)	5 (3.1–9.6)	0.0013**

*Data reported as median with 25–75% interquartile range (IQR). FEV_1_, forced expiratory volume in 1 s; TGA, total gelatinolytic activity; ns, not significant*.

*^a^Based on primary data obtained in Figure S4 in Supplementary Material and Ref. (29)*.

^b^Data presented as relative amounts as determined by densitometry analysis of Western-blot images (Figures S4B,C in Supplementary Material). *P < 0.05, **P < 0.01, and ***P < 0.001, as determined by Kruskal–Wallis test.

### Comparison of SLPI Digestions by Neutrophil Proteases and Other Metalloproteases

**Figure 6:**
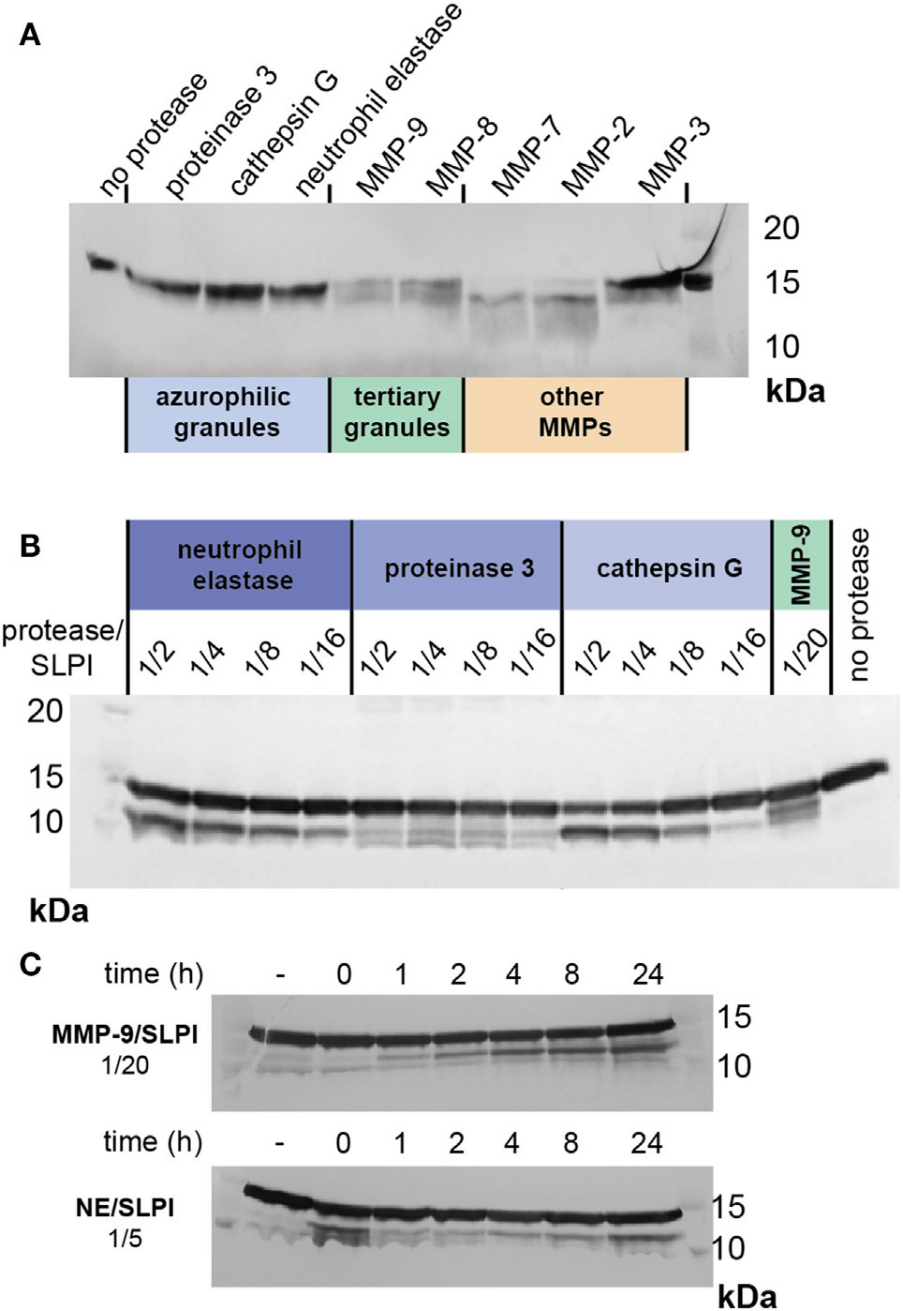
Cleavage of secretory leukocyte peptidase inhibitor (SLPI) by neutrophil proteases and MMPs. **(A)** SDS-PAGE analysis of SLPI digestion by serine proteases present in neutrophil azurophilic granules, MMP-9, and MMP-8 from neutrophil specific and tertiary granules and other MMPs. Digestions performed at protease/substrate ratios of 1/20 and for 24 h at 37°C. Representative image of two experiments. **(B)** SDS-PAGE analysis of SLPI digestion by serine proteases at high protease/substrate ratios in comparison with digestion by MMP-9 at a molar protease/substrate ratio of 1/20. Incubation at 37°C for 24 h. Representative image of two experiments. **(C)** SDS-PAGE analysis of time-dependent SLPI digestion by neutrophil elastase and MMP-9 at protease/substrate ratios of respectively 1/5 and 1/20. Representative image of two experiments.

In conditions such as bronchiectasis, various proteases are released by activated neutrophils, depending on their cellular location and the magnitude of the inflammatory stimulus (45). The three main neutrophil serine proteases are proteinase 3, cathepsin G, and NE, which belong to the azurophilic granules or primary granules. In contrast, MMP-8 (collagenase) and MMP-9 belong to specific and tertiary granules (46). To further characterize the susceptibility of SLPI to degradation by neutrophil proteases (in particular, serine proteases and MMPs), we incubated SLPI with active proteases under similar condition (1/20 protease/SLPI, 24 h at 37°C) (Figure 6A). None of the serine proteases were able to cleave SLPI. Interestingly, cleavages of SLPI by MMP-9 and MMP-8 were similar, while other MMPs cleaved SLPI either differently (MMP-7 or MMP-2) or less efficiently (MMP-3), underlining the importance of MMPs in the generation of cleaved SLPI, even beyond neutrophil biology. Finally, since cleavage of SLPI by NE has previously been reported at high protease/substrate ratios (17), and since NE activity is clearly high in comparison to that of MMP-9, we next evaluated SLPI cleavage with higher serine protease concentrations (Figure 6B). While proteinase 3 hardly resulted in SLPI cleavage fragments, both NE and cathepsin G were able to cleave SLPI at higher concentrations. Finally, we also evaluated the time-dependent cleavage of SLPI by NE (Figure 6C). While MMP-9 again efficiently generated the SLPI** fragment, NE (at a higher concentration) was able to generate a first cleavage at early time-points.

## Discussion

Chronic inflammatory diseases, including many lung pathologies such as bronchiectasis (29), are associated with abundant neutrophil infiltrations. Once activated, neutrophils secrete a series of proteolytic, bactericidal, and immunomodulatory proteins which may contribute to the progression of inflammation and tissue destruction. Previous research had a focus on the destructive effects of neutrophil proteases including serine proteases (NE and cathepsin G) and (MMP-9 and MMP-8) on local tissues (25, 29, 47). One molecule which tempers serine protease activity within tissues is antileukoproteinase or SLPI. In the lungs, SLPI is produced locally by tracheal, bronchial, bronchiolar, and type II alveolar cells, and this basal expression is not linked to MMP-9 (Figure S7 in Supplementary Material). Upon stimulation, SLPI is also secreted by infiltrating immune cells, including monocytes, macrophages, neutrophils, basophils, and eosinophils (3). Previous studies have shown that serine proteases are able to intensify MMP activity, for instance, NE-mediated activation of proMMP-9 (25) and inactivation of the MMP-9 inhibitor TIMP-1 (24). For the first time, we show that MMP-9 is able to cleave SLPI. One cleavage site is situated in the SLPI anti-proteolytic domain, thereby abrogating serine protease inhibition. Interestingly, a first link between MMPs, SLPI, and serine proteases was made over 10 years ago. By high-throughput degradomics analysis of MMP-14 transfected breast carcinoma cells, Tam et al. found SLPI to be one of several new MMP-14 substrates. The authors concluded that C-terminal cleavage of SLPI by MMP-14 may release SLPI from association with cell player proteins (48).

Besides having anti-proteolytic activity, SLPI has been attributed various immunosuppressive functions (9–11). Whereas, we show that MMP-9 is able to reduce LPS-binding activity and anti-inflammatory responses of SLPI in human monocytes, one may wonder how this observation extends toward other specialized SLPI functions. For example, SLPI has been implicated as an essential molecule in granulocytic differentiation (49) and is also involved in immunoglobulin class-switching (50) and other B-cell functions (8). Interestingly, these recently discovered functions also point toward an important intracellular role for SLPI. In view of the recent discoveries of intracellular MMP functions (51), one might speculate whether SLPI is also an intracellular target for MMPs.

**Figure 7:**
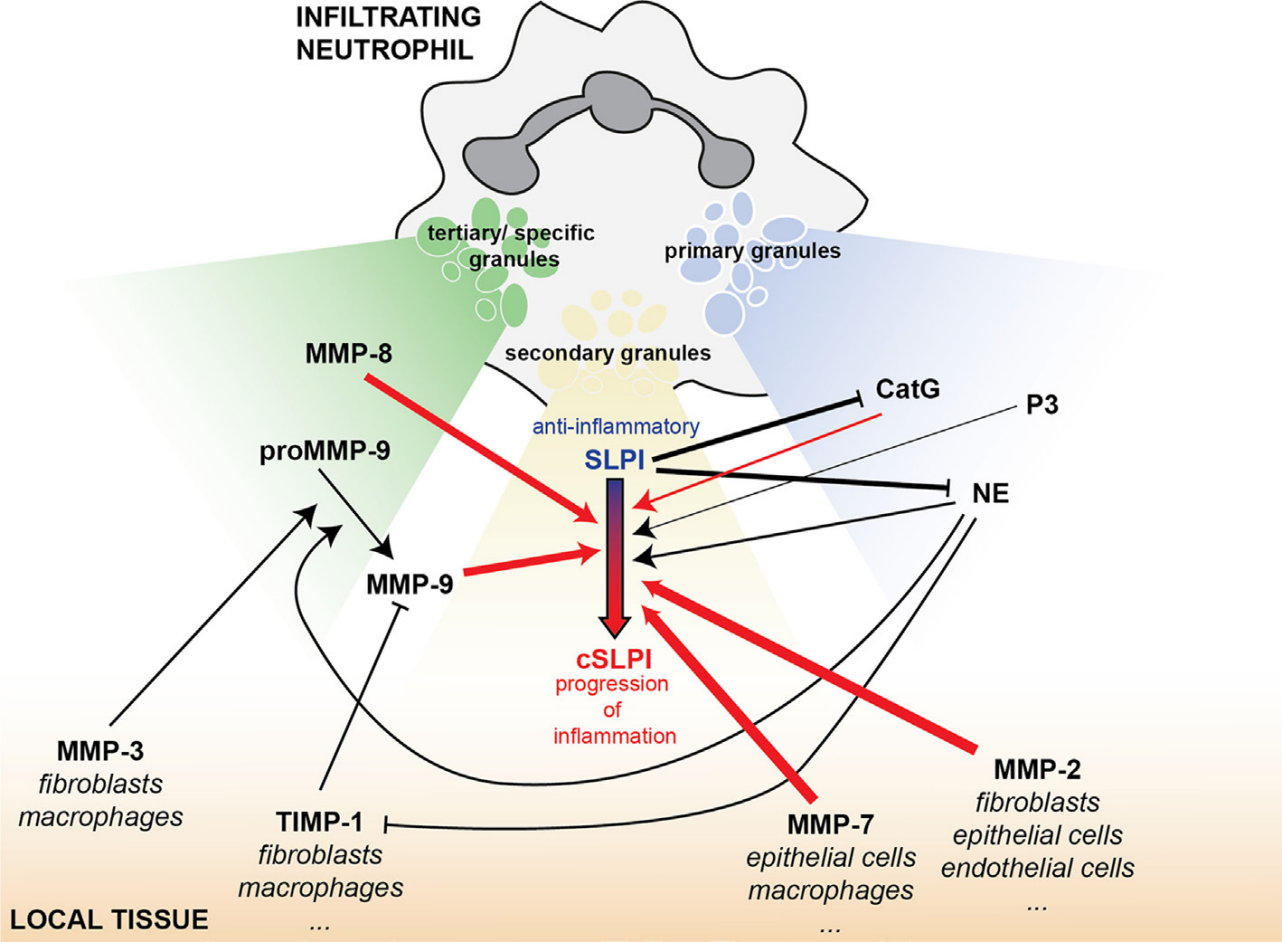
SLPI as an element of the interconnected protease network, bridging MMPs and serine proteases in inflammation. Infiltrating neutrophils carry a battery of proteolytic or bactericidal proteins ready for release upon an inflammatory insult. Besides local mucosal SLPI production, SLPI is also found in the secondary granules of neutrophils (yellow). MMP-9 and MMP-8 are stored in tertiary or specific granules (green) and the serine proteases cathepsin G (CatG), proteinase 3 (P3), and NE in primary granules (blue). Depending on the magnitude of the inflammatory stimulus, tertiary granules are released first, followed by secondary granules and primary granules. This overview shows the interconnectivity between proteases and protease inhibitors released from these vesicles and present in the local tissues (orange). Arrows indicate a positive/stimulatory effect, while solid lines with blunt ends correspond to inhibitory reactions. Line thickness is relative to the efficiency of the inhibitory/stimulatory effect. Red arrows indicate newly described links within this manuscript.

To avoid structural damage, the body meticulously controls proteolytic events (27). This is exemplified not only by the sheer complexity of substrate-protease-inhibitor interactions but also by spatial and temporal separation of these molecules. In neutrophils, SLPI is present in secondary granules from myelocyte stage throughout maturation (52). In contrast, serine proteases, which are targets for SLPI anti-proteolytic activity, belong to different subsets of primary granules (53) and MMP-9 and MMP-8 are found in tertiary granules (45). With tertiary granules (MMP-9 and -8) being secreted first, followed by secondary (SLPI) and finally primary granules (serine proteases), this timing sequence might be crucial to determine the anti-inflammatory and anti-proteolytic status of SLPI. In addition, the proteolytic status of the local environment, prior to release of neutrophil content, might also contribute to the pathophysiological functions of SLPI (Figure 7). In lower airway secretions from cystic fibrosis patients, SLPI levels decrease to 30 nM while NE and MMP-9 concentrations increase up to, respectively, 10–48 nM and 7–15 nM, as determined by immunoassays (30, 31). This corresponds to an NE/SLPI ratio of 1/3 to almost 2/1 and an MMP-9/SLPI ratio of 1/2–1/4. These ratios are in line with the ratios at which we see cleavage fragments for both MMP-9 and NE (Figures 2A and 6B). In combination with our data, showing SLPI cleavage in lower airway secretions from bronchiectasis patients with high MMP-9 and NE content and activity, this indicates that NE and MMP-9 most likely collaborate to achieve SLPI inactivation, followed by increased proteolysis, tissue damage, and inflammation. Our insights contribute to an improved understanding of the protease web with the ultimate hope to identify true target MMPs or MMP-mediated functions and pathways. Our study shows a complex, highly interconnected, and time-dependent interaction between SLPI, MMPs, and serine proteases, which might either contribute to inflammation progression or resolution.

## Ethics Statement

This study was carried out in accordance with the recommendations of the ethical committee for research at UZ/KU Leuven, CME. The protocol was approved by the CME under license (B51060-B32220084152). All subjects gave written informed consent in accordance with the Declaration of Helsinki.

## Author Contributions

JV designed and performed the experiments, analyzed the data, and wrote the manuscript. PG collected and processed patient samples. LB, VR, and EU-B provided help with experiments and writing the manuscript. PP performed protein sequencing. GO and AE-A supervised the experiments and discussed the results. All authors contributed to the writing of the final version of the manuscript.

## Conflict of Interest Statement

The authors declare that the research was conducted in the absence of any commercial or financial relationships that could be construed as a potential conflict of interest.
