# Supplementary material for: Neutrophils and Activated Macrophages Control Mucosal Immunity by Proteolytic Cleavage of Antileukoproteinase
Source: Front Immunol. 2018 May 28;9:1154. doi: 10.3389/fimmu.2018.01154 (PMC5985294; doi:10.3389/fimmu.2018.01154)
Supplement: Supplementary file 1 [file data_sheet_1.docx]

Supplementary Material

Neutrophils and Activated Macrophages Control Mucosal Immunity by Proteolytic Cleavage of Antileukoproteinase

Jennifer Vandooren*, Pieter Goeminne, Lise Boon, Estefania Ugarte-Berzal, Vasily Rybakin, Paul Proost, Ahmed M. Abu El-Asrar, Ghislain Opdenakker*

*** Correspondence:** Jennifer Vandooren: jennifer.vandooren@kuleuven.be

Ghislain Opdenakker: ghislain.opdenakker@kuleuven.be


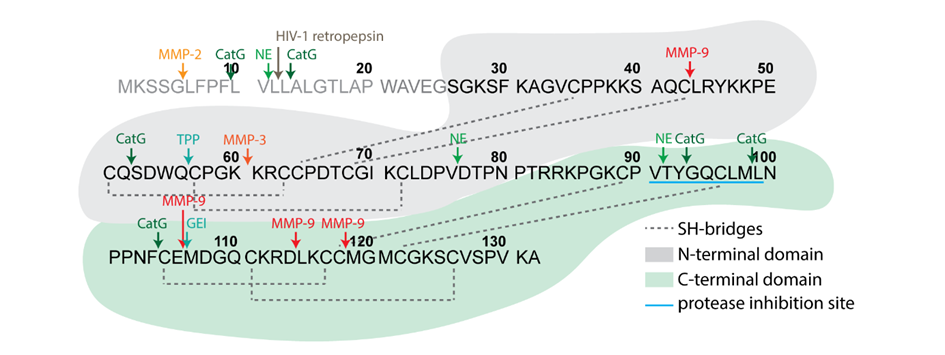


**Supplementary Figure 1.** Schematic representation of human SLPI (GenBank: CAA28187.1) structure, protein sequence and protease cleavage sites on SLPI as predicted by PROSPER. SLPI contains two structurally similar domains, here colored in gray (N-terminal domain) and green (C-terminal domain) backgrounds. The C-terminal domain contains the main amino acid sequence for SLPI protease inhibition properties (sequence VTYGQCLML with blue underlining). Predicted protease digestion sites are indicated by arrows and corresponding proteases marked above. The signal peptide sequence residues 1 to 25 are in grey. CatG, cathepsin G; GEI, glutamyl endopeptidase I; NE, neutrophil elastase; TPP, thylakoidal processing peptidase.

**
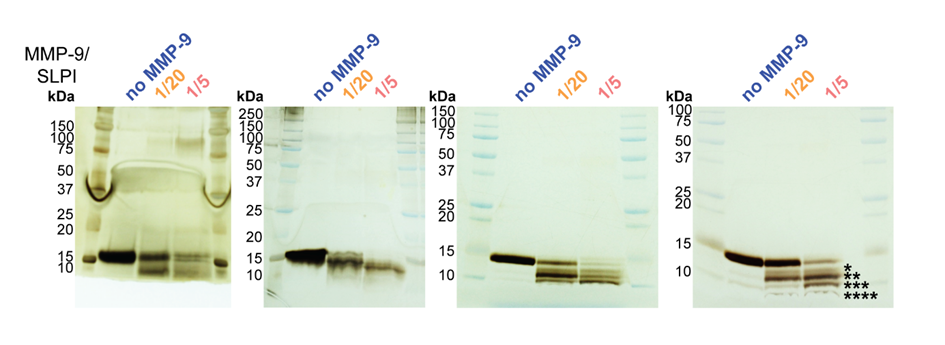
Supplementary Figure 2.** SDS-PAGE analysis of all batches of SLPI and MMP-9-treated SLPI used across experiments. Although some variability exists in the disappearance of intact SLPI, the 1/20 batch has mainly fragments with C-terminally cleaved SLPI (SLPI**) while the 1/5 batch contains more SLPI with both N-terminal and C-terminal cleavage (SLPI***).

**
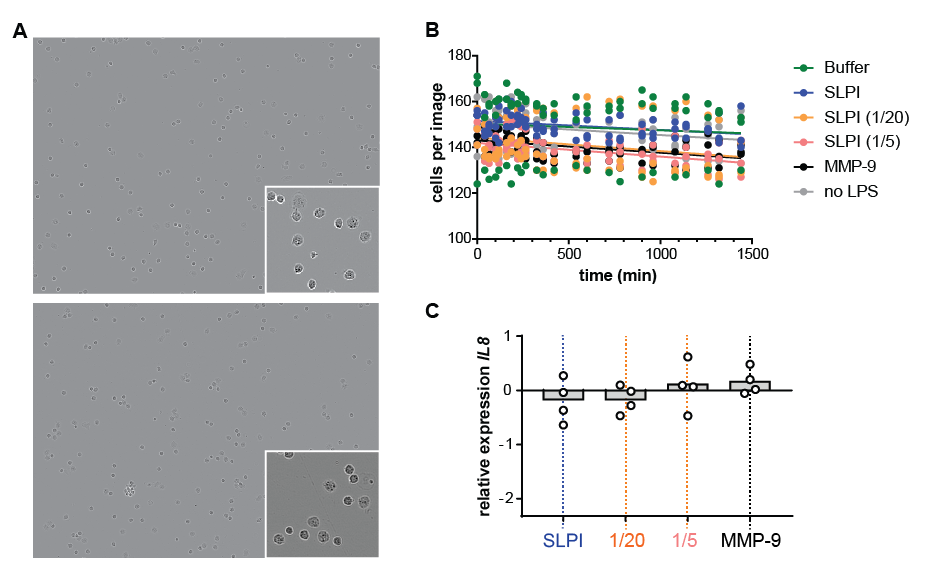
**

**Supplementary Figure 3.** Human monocytes images and cell counts. (**A)**, representative images of monocytes after purification by anti-CD14 selection. The inserts illustrate the morphology of about 10 cells at higher magnification. (**B)**, Counts of cells per image. Each sample represents the average of three images per well and each condition consists of triplicates (n = 3). No significant differences were observed between cell counts. (**C**), analysis of *IL8* RNA expression in human monocytes treated with SLPI or cleaved SLPI (1/5 and 1/20) prior to stimulation with LPS (1 µg/ml). Each data point represents an independent experiment with a fresh and different pool of human monocytes. Data represented as log2 fold change compared to cells stimulated only with LPS.

**
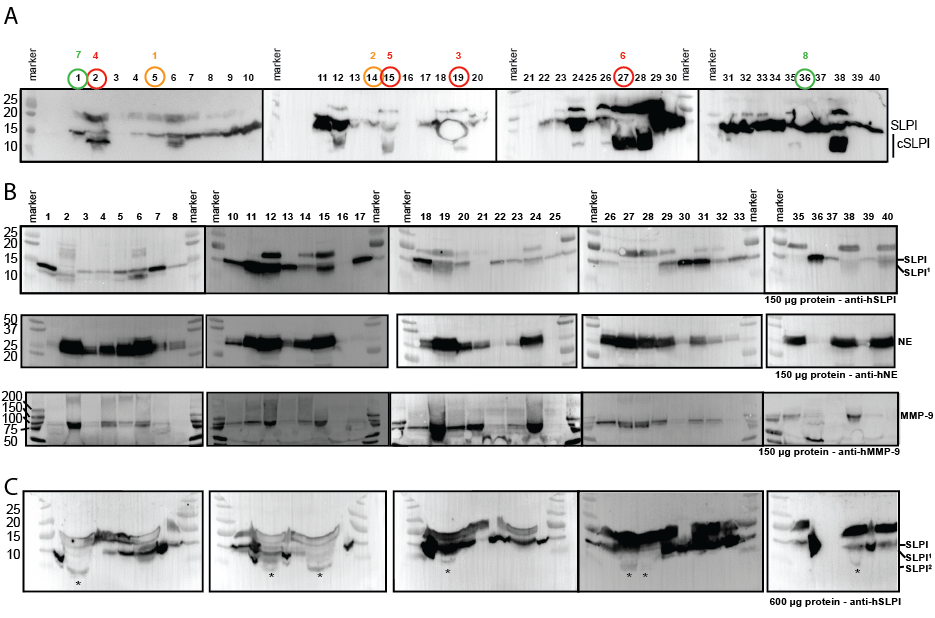
**

**Supplementary Figure 4.** (**A**), Detection of SLPI and cleaved SLPI (cSLPI) in sputum samples from patients with bronchiectasis by Western blot analysis of 30 µl sputum sample. Eight samples shown in panel A are circled and divided into three categories: strong SLPI signal and no cSLPI (green, circles and numbers), weak SLPI signal but no cSLPI (orange, circles and numbers) and a weak signal for intact SLPI and visible cSLPI (red, circles and numbers). Numbers indicated above the selected samples indicated loading order of these samples in Figure 5. (**B**), Western-blot images of sputum samples corresponding to 150 µg total protein. Western-blot were developed with, respectively, anti-SLPI, anti-NE and anti-MMP-9 antibodies. (**C**), Western-blot images of sputum samples corresponding to 600 µg total protein and developed with anti-SLPI antibodies.


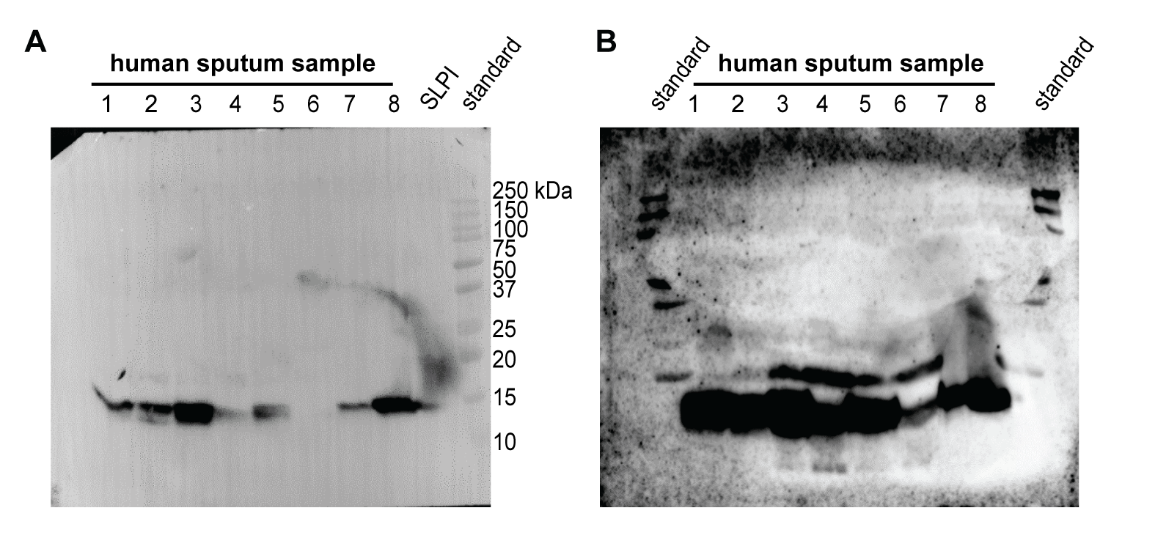


**Supplementary Figure 5.** Full view of Western blot images used for Figure 5. Western blot analysis of sputum samples from eight selected (see **Supplementary Figure 4**) bronchiectasis patients probed with anti-SLPI. **(A)**, total loading volume of each sample corresponds to 150 µg total protein. **(B)**, total loading volume corresponds to 600 µg total protein.


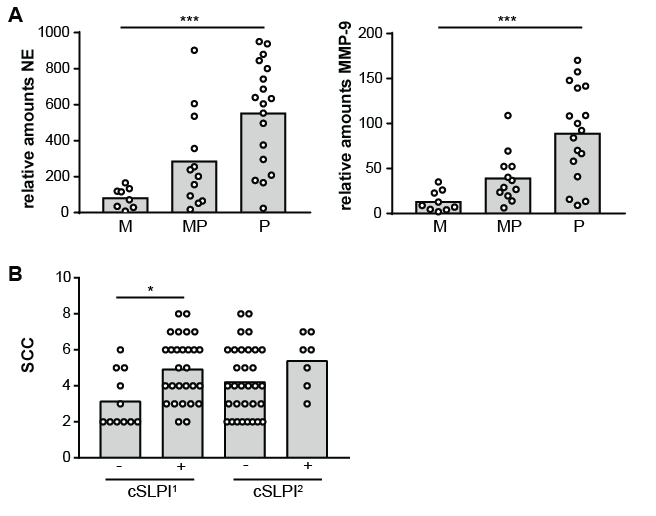


**Supplementary Figure 6.** (**A**) Relative amounts of NE and MMP-9 (determined by Western blot), in function of sputum purulence. (**B**) Sputum color chart (SCC) scores in function of the presence or absence of the indicated SLPI cleavage fragments (cSLPI^1^ and/or cSLPI^2^). *P<0.05, ***P<0.001, as determined by Kruskal-Wallis test.


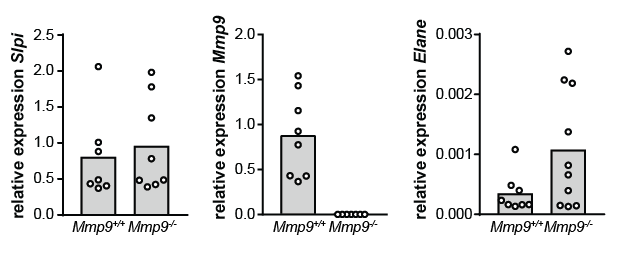


**Supplementary Figure 7.** RT-qPCR analysis of *Slpi* (Mm.PT.58.30450882), *Mmp9* (Mm.PT.58.45834688.g) and *Elane* (neutrophil elastase, Mm.PT.58.6682392.gs) in lung tissue of wild-type (*Mmp9^+/+^*) versus *Mmp9* deficient (*Mmp9^-/-^*) mice. Data are shown as relative expression compared to *Tbp* (Mm.PT.58.17504874). All procedures were conducted in accordance with protocols approved by the local Ethics Committee.

**Supplementary Table 1**. Patient characteristics of subset from Ref. 29.

| Age (years) | 63 (38-73) |
| --- | --- |
| Gender (% male/female) | 55/45 |
| FEV_1_ (%) | 64 (52-87) |
| FVC (%) | 82 (61-99) |
| LCQ total score | 15 (10-18) |
| Total modified Brody score | 19 (14-26) |
| SCC (% M/MP/P) | 18/33/49 |

Data reported as median with 25-75% interquartile rage (IQR). FEV_1_, forced expiratory volume in 1 s; FVC, forced vital capacity; LCQ, Leicester Cough Questionnaire (1); SCC, Sputum Colour Chart (2); M, mucous; MP, mucopurulent; P, purulent.

1 Birring SS, Prudon B, Carr AJ, Singh SJ, Morgan MD, Pavord ID. Development of a symptom specific health status measure for patients with chronic cough: Leicester Cough Questionnaire (LCQ). Thorax (2003) 58(4):339-43.

2 Murray MP, Pentland JL, Turnbull K, MacQuarrie S, Hill AT. Sputum colour: a useful clinical tool in non-cystic fibrosis bronchiectasis. Eur Respir J. (2009) 34(2):361-4.
